# Transcranial Doppler Ultrasonography as a Diagnostic Tool for Cerebrovascular Disorders

**DOI:** 10.3389/fnhum.2022.841809

**Published:** 2022-04-29

**Authors:** Yuanmei Pan, Wenbin Wan, Minjie Xiang, Yangtai Guan

**Affiliations:** Department of Neurology, Ren Ji Hospital, School of Medicine, Shanghai Jiao Tong University, Shanghai, China

**Keywords:** blood flow, cerebrovascular disease, neurological condition, non-invasive, TCD

## Abstract

Imaging techniques including transcranial Doppler (TCD), magnetic resonance imaging (MRI), computed tomography (CT), and cerebral angiography are available for cerebrovascular disease diagnosis. TCD is a less expensive, non-invasive, and practically simpler approach to diagnosing cerebrovascular disorders than the others. TCD is a commonly available and inexpensive diagnostic tool. However, owing to its large operator dependency, it has a narrow application area. Cerebrovascular disease indicates a group of disorders that alter the flow of blood in the brain. The brain’s functions can be temporarily or permanently impaired as a result of this change in blood flow. Timely diagnosis and treatment can restore the brain-impaired functions, resulting in a much-improved prognosis for the patients. This review summarizes the basic principles underlying the TCD imaging technique and its utility as a diagnostic tool for cerebrovascular disease.

## Introduction

### Transcranial Doppler Ultrasonography

**FIGURE 1:**
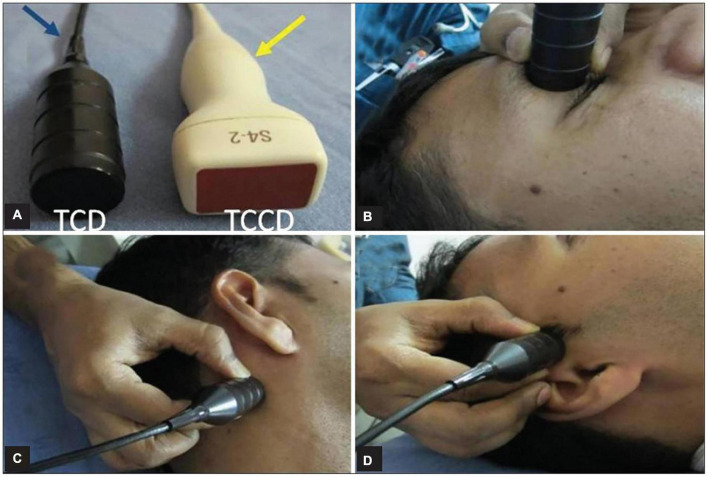
Panel **(A)** indicates ultrasonic probes, with blue arrows indicating TCD and yellow arrows indicating Transcranial color-coded sonography (TCCS). Panels **(B–D)** show transcranial acoustic windows used in TCD evaluation, including transorbital, suboccipital, and transtemporal windows, accordingly (Bathala et al., 2013).

In the basal cerebral arteries, the TCD imaging tool uses low-frequency ultrasonic waves (i.e., ≤2 MHz) to evaluate blood flow parameters and cerebrovascular hemodynamics in real-time. Such parameters provide physiologic information that can be used in combination with structural information taken from a variety of existing imaging techniques (Purkayastha and Sorond, 2012). When dealing with cerebrovascular complications, TCD is considered to be the most practical technique to keep track of vascular alterations in response to treatment. TCD’s ease of use as a diagnostic approach could lead to an increase in its utility in clinical and research settings for a variety of cerebrovascular conditions (Bhogal, 2021). It is only feasible to insonate the cerebral arteries *via* the acoustic window while using a low-frequency probe (Basri et al., 2021). In adults, the four most frequently used acoustic windows are temporal, suboccipital, orbital, and submandibular. Figure 1 illustrates a variety of acoustic windows.

The anterior, middle, and posterior windows make up the transtemporal window. When the intracranial carotid artery (ICA) bifurcation terminates in the anterior, middle, and posterior cerebral arteries (abbreviated as ACA, MCA, and PCA, accordingly), it can be identified at depths of 55–65 mm with the simultaneous flow toward or outward from the probe. The transtemporal window can also insonate the ICA bifurcation at the underlined depths with flow (Fodale et al., 2007; Purkayastha and Sorond, 2012). The ophthalmic artery (OA) and carotid siphon can be evaluated through the transorbital window. The vertebral and basilar arteries can be insonated using a flexed neck and the suboccipital window. In the flexed neck, the submandibular window can be used to detect the distal ICA at a 40–60 mm depth (Alexandrov et al., 2011). TCD guidelines for clinicians were published by the American Academy of Neurology which have been shown in Table 1 (Sloan et al., 2004).

**TABLE 1:** Guiding principle for normal TCD evaluations.

Artery	Window	Depth (mm)	Direction	MFV (cm/s)
ACA	Temporal	60–85	Away	50 ± 11
BA	Occipital	80–110	Away	41 ± 10
ICA	Orbital	60–80	Bidirectional	45 ± 15
MCA	Temporal	30–60	Toward probe	55 ± 12
OA	Orbital	40–60	Toward	20 ± 10
PCA	Temporal	60–70	Bidirectional	40 ± 10
VA	Occipital	60–80	Away	38 ± 10

As indicated in Table 1, each vessel has a unique depth range, flow direction, and appropriate age-associated flow velocity (FV) range; nevertheless, these data are influenced by a variety of physiological and pathological conditions, such as increase in age, CSF pressure, and central venous pressure lead to the decrease in the FV. While increased blood viscosity results in the elevation in FV. Moreover, vasoactive medications including drugs with vasodilatation and vasoconstriction properties results in the increase and decrease in FV (Kassab et al., 2007).

**FIGURE 2:**
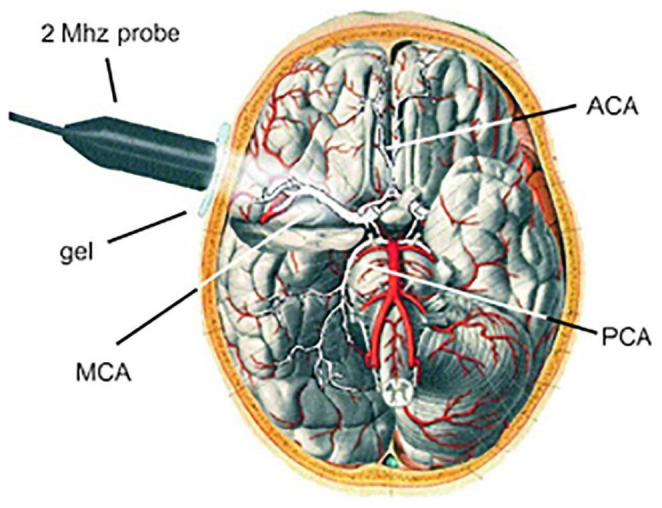
Measurement of the cerebral blood flow velocity (CBFV) in the MCA by TCD. The Circle of Willis and the MCA can be seen when the 2 MHz probe is positioned transtemporally. The ACA and PCA can be insonated by TCD as well (D’Andrea et al., 2016a).

A thorough TCD evaluation needs to include measurements from each of the four windows, and the path of blood flow within every main branch of the circle of Willis (Figure 2) should indeed be examined, despite the fact that each window has distinct advantages for particular arteries. In this view, TCD revolves around the Circle of Willis (Spencer and Whisler, 1986). For the TCD scan, the ultrasound transducer is first placed on the temporal bone, over the closed eyelid, and on the base of the skull to capture the signals. The ACA, MCA, and PCA, the ophthalmic and carotid siphons, and the vertebral and basilar arteries are among the arteries that can be investigated (D’Andrea et al., 2016b).

### Basic Principles of Transcranial Doppler

This review does not cover all of the concepts and interpretations of the TCD results, however, we did provide an overview. TCD ultrasonography is based on the Doppler effect and the Bernoulli principle, which uses sound waves to move more quickly through the body. Transcranial ultrasonic waves from the Doppler probe reflect off of circulating RBC’s in intracerebral vessels. The “Doppler shift frequency,” or variation in frequency between emitted and reflected waves, is directly proportional to the velocity of RBC’s circulation (BFV). The recorded Doppler signals may be a mixture of distinct Doppler frequency shifts, which results in a spectrum showing on the TCD monitor of individual RBC’s flow due to the laminar blood flow (DeWitt and Wechsler, 1988). The Doppler effect and the Doppler equation can be used to determine variations in blood velocity (vasospasm detection) (Nicoletto and Burkman, 2009).

The BFV and some other properties of flow within the insonated blood vessels can then be measured using spectral analysis. When performing this spectrum analysis, the researchers were able to derive several important parameters, including the mean systolic velocity, peak systolic velocity, pulsatility index, and end-diastolic velocity (abbreviated as V_mean_, VS, PI, and EDV, accordingly) (Harders, 2012). The V_mean_ is a time-dependent continuous trace of peak velocities that are computed and displayed automatically on most TCD monitors (Uppal and Mogra, 2010). The following formula explains the connection between FV (reflector speed) and Doppler shift frequency.


Reflectorspeed(cm/s)=(DopplerShift) ×propagationspeed/2×Incidentfrequency×cos(θ)

A wave’s propagation speed is a constant that can be calculated for different mediums (the speed in soft tissue is 1541 m/s). Theta (θ) is the angle of insonation, or the angle of the emitted wave, with respect to the vessel’s direction (blood flow). When the angle is zero, or the emitted wave is parallel to the flow direction, the cosine of zero equals one, and we get the most accurate measure of FV (Nicoletto and Burkman, 2009). The larger the angle, the greater the angle’s cosine, and hence greater the error in our velocity measurement. As a result, to keep the inaccuracy below 15%, this angle should be kept to less than 30°.

### Frequently Used Terminologies in Transcranial Doppler Imaging

**FIGURE 3:**
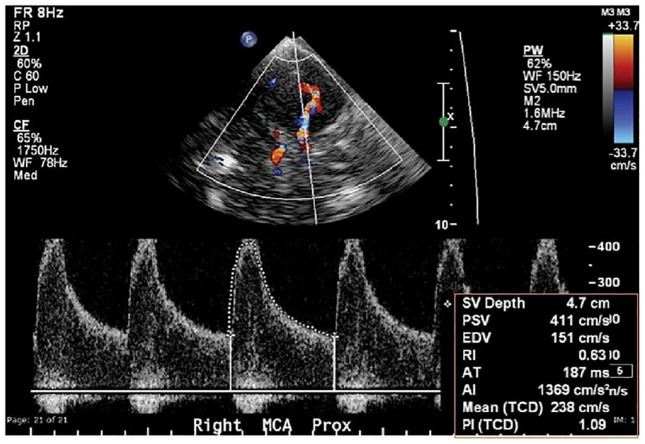
Typical transtemporal window parameters used in TCD imaging (Naqvi et al., 2013).

The following terminologies are frequently used in TCD imaging (Figure 3).

#### Peak Systolic Velocity

Peak systolic velocity is a spectral Doppler index measured by Doppler ultrasonography. This is the initial peak on each cardiac cycle’s TCD waveform (Uppal and Mogra, 2010; Berry et al., 2021).

#### End-Diastolic Velocity

The EDV is a spectral Doppler index that indicates a low resistance cerebral artery flow pattern in all main intracranial arteries, ranging from 20 to 50% of the PSV (Altinbas et al., 2014; Oglat et al., 2018).

#### Mean Flow Velocity

The MFV is calculated as EDV plus one-third of the difference between PSV and EDV (Sun et al., 2018).

#### Pulsatility Index

Pulsatility Index is commonly used to measure flow resistance, which is determined by subtracting EDV from PSV and dividing the resulting value by MFV. PI is the most common TCD parameter for determining flow resistance, i.e., PI values greater than 1.2 represent high resistance blood flow (Bellner et al., 2004).

#### Resistance Index

The RI is a TCD parameter that is used to measure flow resistance. Flow resistance proximal to the point of insonation is represented by this parameter. The RI is determined by subtracting EDV from PSV and dividing the value by PSV. If the RI value is less than 0.75, it is considered normal (Ke et al., 2020).

#### Systolic Diastolic Ratio

The percentage difference between PSV and EDV is known as the systolic diastolic ratio (S/D). It is not implemented in any clinical setting (Akaishi et al., 2020).

#### Heart Rate

Transcranial Doppler spectra typically include HR as an essential component because it can affect a variety of flow parameters (Mitra et al., 2014).

#### Types of Transcranial Doppler Devices

Transcranial Doppler devices, both non-duplex and duplex, are currently available. Non-duplex TCDs are non-imaging, whereas duplex TCDs are imaging. The arteries are detected “blindly” in non-duplex systems using the spectrum display and audible Doppler shift. The cranial window, sample volume depth, probe orientation, blood flow direction, link to the terminal internal carotid artery, and response to various actions such as compression of the common carotid artery and eye opening and closing are utilized to identify particular vessels (Bhogal, 2021).

The imaging B-mode transcranial color-coded duplex (TCCD) combines pulsed wave Doppler ultrasonography with a cross-sectional picture of the insonation area, allowing the arteries to be identified in relation to various anatomic locations. While recording blood flow velocities, the color-coded Doppler also indicates the flow direction in respect to the probe (transducer) (Krejza and Baumgartner, 2004). An advanced technology known as the power motion-mode TCD (PMD/TCD) has now become developed, which displays multi-gate flow information in the power M-mode display at the same time. To display flow signals simultaneously, it uses numerous overlapping sample volumes. PMD/TCD appears to make TCD handling easier by enabling the temporal window position and alignment of the incident signal, allowing for CBFV measurements through various arteries. However, these imaging TCD techniques improve TCD performance by taking into account the angle of the insonation. There are still clinical applications for the more recent imaging modalities that are still being developed. Duplex TCD technologies will likely replace non-duplex TCD in clinical practice as time goes on (Purkayastha and Sorond, 2012).

### Transcranial Doppler Utility in Cerebrovascular Disorders

**FIGURE 4:**
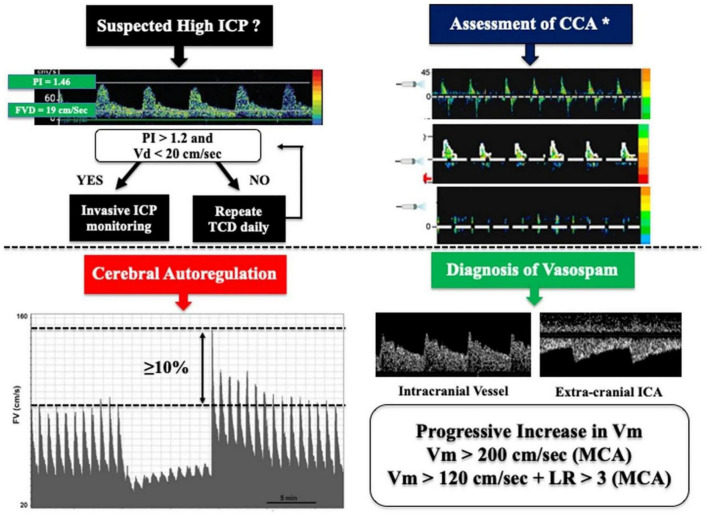
In clinical practice, simplified algorithms for evaluating brain death, intracranial hypertension, cerebral vasospasm, and autoregulation using TCD. PI indicates pulsatility index while Vd, Vm, and Vs indicate diastolic flow, mean flow, and systolic flow velocity, accordingly (Robba and Taccone, 2019). While LR represent the lindegaard ratio, and CCA represent cerebral circulatory arrest. * The three reported images represent reverberating flow (top), systolic pikes (middle), and no flow (bottom), respectively (Robba and Taccone, 2019).

Transcranial Doppler is thought to be effective in diagnosing cerebrovascular diseases including cerebral sinus venous thrombosis (CSVT) (Hsu et al., 2004), acute ischemic stroke (AIS) (Shahripour et al., 2021), intracranial atherosclerotic stenosis (ICAS) (Felberg et al., 2002), and cerebral vasospasm (CV) (de Carvalho Nogueira et al., 2021). TCD’s sensitivity and specificity are considered lower than other imaging modalities including CTA and MRA, but it can detect the majority of lesions amenable to interventional treatment, such as stenting and local thrombolysis as well as angioplasty, and can detect as well as monitor the impact of the underlined interventions. TCD can evaluate the cerebral hemodynamic implications of steno-occlusive lesions in the cervical parent artery and to monitor cerebral perfusion pressure, intracranial pressure (ICP), and midline deviation at the patient’s bedside, allowing us to explore the patient’s neurocritical pathological features in a non-invasive manner (Caballero-Lozada et al., 2021). Validation of brain death by showing cerebral circulatory arrest is another hallmark feature of TCD (Akif Topcuoglu, 2012). The underlined data has been represented in Figure 4. We discussed herein the use of TCD in cerebrovascular diseases.

### Acute Ischemic Stroke

Ischemic stroke occurs when blood flow to a portion of the brain is suddenly interrupted, leading to the loss of neurological function. AIS, which is more common than hemorrhagic stroke, is triggered by embolic or thrombotic occlusion of a cerebral artery (Huang et al., 2018).

Transcranial Doppler was utilized to diagnose forty-eight individuals with AIS in the MCA area after intravenous thrombolytic therapy. When the patient was in the supine posture for the TCD evaluations (portable DWL), MCA (M1 branch) blood flow was observed (depth ranging from 40 to 60 mm) using a 2 MHz probe (left and right sides) from both temporal windows. The peak systolic (72.2 cm/s), end-diastolic (27.8 cm/s), and mean BFV (38.7 cm/s) of the MCA (M1 branch) were all measured, followed by calculating the PI (1.1) (Uzuner et al., 2013). The results showed that among the blood flow parameters evaluated by TCD as a modest way of assessment in AIS patients, PI may contributes in predicting a positive functional as well as clinical prognosis post thrombolytic therapy (Allendoerfer et al., 2006).

Alexandrov et al. studied TCD as a diagnostic tool for acute cerebral ischemia in 130 patients. Digital subtraction angiography (DSA), MRA, and CTA were used to determine the specificity, sensitivity, and overall correctness of TCD results. At a 15% rate of missing temporal windows, TCD demonstrated an accuracy of 88% for aberrant (occlusion and stenosis) vs. normal arteries, with a positive and a negative predictive value of 87.5 and 88.6%, accordingly. They also found that lesions in the MCA area had 88.6% specificity. However, according to another study, total TCD sensitivity and specificity were found to be 96 and 33% in evaluating aberrant CBFV in damaged vessels (posterior as well as anterior circulation) in the first 24 h post-AIS. The reduced specificity could be attributed to occlusion of intracranial arteries or extracranial illness, as Camerlingo et al. (1993) reported a specificity of approximately 92% for TCD (Burgin et al., 2000; Alexandrov et al., 2004). Although the above results showed the fluctuation in specificity as well as sensitivity of TCD between MCA area and affected vessels (Demchuk et al., 2000), quantitative CBFV evaluations using TCD might be a helpful technique for selecting patients for reperfusion therapy as compared to other techniques including MRI.

While performing a rapid TCD in AIS, clinical stroke localization should be considered. TCD should begin in the predicted normal hemisphere to develop an understanding of the normal artery waveform pattern and velocity distribution, as well as the expected temporal acoustic window reliability (El Sayed et al., 2021). Because TCD has high diagnostic accuracy in AIS (Bhattacharya et al., 2021), it should be conducted soon after the onset of symptoms. TCD has sensitivity, specificity, positive, and negative predictive values of 96, 75, 96, and 75%, accordingly, for evaluating an offending lesion in AIS. However, 94, 90, 94, and 90%, accordingly, are seen in carotid duplex sonography. The underlined data suggested that when TCD is used in combination with other techniques, the diagnostic yield is higher (Yeo and Sharma, 2010).

### Intracranial Atherosclerotic Stenosis

One of the most prevalent causes of stroke globally is ICAS of a major intracranial artery, which is linked with an increased risk of recurrent stroke relative to other stroke subtypes (Gorelick et al., 2008). TCD is a less expensive, non-invasive, accessible, and technically simpler diagnostic technique for ICAS (Hussain and Gupta, 2008; Leng et al., 2014). Finding out about ICAS is beneficial since, unlike static imaging modalities like CTA or MRA, it delivers real-time information on BFV (Feldmann et al., 2007; Guan et al., 2013). TCD is one of the most commonly utilized tests because of its ease of use and low cost; also, it allows for bedside monitoring in crucial situations (Alexandrov et al., 1999). It also gives real-time information on the hemodynamics of the intracranial circulation. TCD can evaluate not only the location of intracranial artery stenosis or occlusion but also the severity of the lesion (Jaiswal et al., 2019).

A case-control study was conducted at Bethesda Hospital in Yogyakarta (Indonesia), with 234 ischemic stroke patients. The researchers looked at the distribution of intracranial artery abnormalities, which were divided into anterior (ACA and MCA) and posterior circulations (vertebral segment and proximal basilar artery). ICAS is prevalent in ischemic stroke patients, according to the reported data (Jaiswal et al., 2019). TCD had been used to detect narrowing or blockage in intracerebral arteries. With a 2-MHz probe, the TDOPTC9000P unit performed TCD tests. The diagnostic criteria for intracranial stenosis were the PSV or MSV. The diagnostic criteria for stenosis in the MCA are PSV ≥ 140 cm/s or MSV ≥ 80 cm/s. The diagnostic criteria in the anterior cerebral artery were PSV ≥ 120 cm/s. While in the posterior circulation, the diagnostic criteria were PSV ≥ 90 cm/s or MSV ≥ 60 cm/s (Sarkar et al., 2007). The proportions of anomalies in ischemic stroke patients were 37% stenosis, 31% atherosclerotic, and 21% hypoperfusion. Only 11% of the participants had normal vascularization. As compared to the posterior circulation (17%), anterior circulation stenosis (63%) was more common, followed by those with both anterior and posterior circulation stenosis (20%) (Pinzon and Wijono, 2021). Because TCD can now safely and consistently detect cerebral occlusive disease, researchers may investigate the frequency of intracranial stenosis in patients with numerous risk factors (Zarei et al., 2010).

Another study compared the precision of TCD to MRA in detecting ICAS in ACI patients. A total of 115 patients i.e., males and females [77 (66.95%) and 38 (33.04%), accordingly] were enrolled in the study. TCD and MRA had a 0.56 and 0.04 agreement in diagnosing stenosis in the anterior (ACA) and the posterior circulation artery (PCA), accordingly (Jaiswal et al., 2019). For the identification of ICAS, the sensitivity, specificity, and positive, as well as negative predictive values for anterior and posterior cerebral artery were 85.9, 90.0, 98.2, and 50.0%, and 73.5, 86.7, 96.2, and 40.0%, accordingly. TCD and MRA had a moderate agreement in detecting anterior circulation stenosis and a fair agreement in detecting posterior circulation stenosis in the investigation of ICAS. The data shown above demonstrated that TCD had a better diagnostic accuracy in the anterior circulation relative to the posterior circulation (Jaiswal et al., 2019).

### Cerebral Sinus Venous Thrombosis

Cerebral sinus venous thrombosis is a rare and complex disorder that affects the dural venous sinus and cerebral veins and has sex-related specific causes (Alvis-Miranda et al., 2013). Central venous thrombosis is the most common risk factor for CSVT. On March 15, 2021, the Paul Ehrlich Institute (Federal Institute for Vaccines and Biomedicines) reported CSVT in seven COVID-19 patients (20–50 years old) with thrombocytopenia following vaccination with AstraZeneca’s COVID-19 vaccine (Cavalcanti et al., 2020; Dakay et al., 2020). It has also been discovered that head and neck infections, as well as severe systemic disorders, are the leading causes of the aforementioned syndrome in youngsters (Ichord, 2017). Because of its variable and non-specific presenting patterns, CSVT can be difficult to diagnose. Early detection and treatment can reduce morbidity and mortality, dramatically improving the prognosis for those who are affected. However, the diagnosis of CSVT is particularly problematic due to the vast range of clinical manifestations. For the diagnosis of affected persons, a high index of probability and good clinical skills are required (Siddiqui and Kamal, 2006). Serial Venous TCCS was presented in a case study as an effective tool to detect disruptions in the cerebral venous circulation and to monitor patients with CSVT. With venous TCCS, valuable information including flow direction and variations in the Doppler flow waveform may be collected simply and non-invasively, which cannot be obtained frequently with time-of-flight MR angiography (Siddiqui and Kamal, 2006). Although TCCS cannot evaluate each cerebral venous structure, it can be used as an adjunct to other examinations by giving hemodynamic data on venous circulation (Zhu et al., 2019).

Zhu et al. (2019) studied the detection rate and diagnostic accuracy of TCCS and CE (contrast-enhanced)-TCCS of cerebral veins and sinuses, as well as the diagnostic accuracy of TCCS for both SS and TS (straight and transverse sinus) thromboses. Relative to the MRI/MRV, CE-TCCS, s sensitivity and specificity for SS thrombosis, right TS thrombosis, and left TS thrombosis was found to be 100 and 96.3%, 100 and 100%, and 100 and 94.4%, accordingly. The obtained data showed a high rate of identification for cerebral veins and sinuses while the diagnostic accuracy was also found to be high for SS and TS thrombosis. The underlined data suggests that it could be used to evaluate SS and TS thrombosis if no other neuroimaging technologies are available, or if the sole alternative is a bedside examination. TCD provides data on venous hemodynamics that is not available from other neuroimaging modalities, and hence could be used to diagnose CSVT.

Furthermore, patients with CSVT may experience headaches, nausea, vomiting, blurred vision, and symptoms of increased intracranial pressure, as well as focal neurological indications that resemble subarachnoid haemorrhage (SAH), or ACI. Additionally, the patient may be unwilling to cooperate, even for a fast test, such as CT; MR imaging and angiography may require anesthesia, making study of these individuals challenging (Khealani et al., 2008). While TCD ultrasonography is a simple approach that can be conducted at the patient’s bedside and is safe to use even in extremely restless patients (D’Andrea et al., 2016a). Additionally, a case study was presented in which patients with sagittal venous sinus thrombosis were treated with TCD ultrasonography through the temporal bone window of the left and right MCA’s at 45 mm or 55 mm depth. According to the findings, strong venous signals paralleling the MCA’s were found, with flow directed toward the center of the head, likely due to enhanced flow *via* collateral venous channels to the deep cerebral veins bypassing the thrombosed sagittal sinus. As the patient improved, the venous signals vanished. These significant venous signals next to the MCA provided the initial indication of the diagnosis, which was later validated by CT. However, it is not claimed that a TCD study gives a conclusive diagnosis, but rather that it may provide sufficient evidence to justify a more aggressive examination to develop a positive diagnosis. Being aware of possible TCD signs in patients with a similar history may aid in the identification of CSVT more quickly (Wardlaw et al., 1994).

### Cerebral Vasospasm

Post Aneurysmal subarachnoid hemorrhage (aSAH), the large and medium intracranial arteries become narrow that resulting in lowering brain perfusion and this condition is called cerebral vasospasm (Diringer et al., 2011). Delayed cerebral ischemia (DCI) triggered by CV has been linked with considerable morbidity and death, hence establishing efficient preventive, diagnostic, and therapeutic approaches for vasospasm has attracted researchers’ attention (Vergouwen et al., 2010; Sun et al., 2018).

In SAH patients, TCD is a non-invasive monitoring method for CV. Although CTA and cerebral DSA are viable alternatives for diagnosing CV, they come with added hazards, are invasive, and involve radiation exposure (Sarkar et al., 2007; Kumar and Alexandrov, 2015). Better imaging of posterior circulation vessels is now possible, especially in patients with thick temporal bone, due to the advent of new technology such as power motion Doppler. Vasospasms are also dynamic in nature, continuously reorganizing the power action. Doppler’s window finding tool visualizes flow intensity, giving it a lead over other traditional approaches (Sarkar et al., 2007). By monitoring trends in both pulsatility and waveform alterations, Doppler ultrasonography can detect other physiologic effects of SAH (such as elevated ICP) can be detected using Doppler ultrasonography (Sloan et al., 1994). Numerous studies demonstrate a link between MCA FVm and the severity of vasospasm in MCA vasospasm including mild (FVm < 120 cm s^–1^), moderate (FVm ranges from ≥120 cm s^–1^ to <200 cm s^–1^), and severe vasospasm (FVm > 200 cm s^–1^ c) (Sloan et al., 1994). Such elevation in FVm can be used to indirectly suggest the occurrence of vasospasm and can be detected with high sensitivity and specificity using TCD up to 2 days ahead of symptom onset, with a positive predictive value of 0.97 (Lysakowski et al., 2001). Vora et al. reported that an FVm of > 120 cm s^–1^ had sensitivity and specificity of 0.72 and 0.88, accordingly (relative to cerebral DSA), in diagnosing MCA vasospasm with 33% stenosis in a retrospective investigation. In the same study, an FVm of <120 cm s^–1^ correlated to a negative predictive value of 0.94 (Vora et al., 1999; Sarkar et al., 2007).

Previous investigations have also explained analogous FVm thresholds for BA vasospasms, although they need the addition of an SR threshold. FVm > 85 cm s^–1^ and SR > 3 were shown to have a sensitivity of 0.92 and specificity of 0.97 in probing BA vasospasms of greater than 50% blockage (Sviri et al., 2006). According to a comparable study, FVm > 95 cm s^–1^ refers to a sensitivity of 1.00 (Sloan et al., 1994). A study reported that patients with an SR > 3 had 50% BA stenosis. To establish a baseline FVm, physical tests and TCD are done every day. Next, MRI perfusion, MRA, CTA, or cerebral DSA were used to validate vasospasm if any anomalies, obscurities, or problems are noticed in the physical exam or TCD, even if it’s at a low threshold (Sviri et al., 2006). Despite the fact that angiography is still the gold standard, TCD has been employed on a regular basis to assess vasospasm, lead further investigations, and follow the treatment. Indeed, cerebral vascular constriction has also been evaluated, which is linked to a progressive elevation in mean FV (Kumar et al., 2016). In the case of clinical suspicion of vasospasm (i.e., neurological impairment), the MCA mFV > 200 cm/s cutoff has been utilized to promptly start therapy and do further diagnostic imaging studies (i.e., cerebral CT perfusion or angiography) (Vora et al., 1999). If the mFV > 120 cm/s and < 200 cm/s, the submandibular window has been used to evaluate the mFV in the extracranial internal carotid artery and evaluate the Lindegaard ratio (LR) to distinguish between vasospasm and cerebral hyperemia (Lindegaard et al., 1989). The sensitivity, specificity, positive, and negative predictive value of TCD for diagnosing vasospasm of MCA was found to be 90% (95% confidence intervals [CIs] 77–96%), 71% (95% CI 51–84%), 57% (95% CI 38–71%), and 92% (95% CI 83–96%), accordingly. However, angiography or cerebral CT perfusion are still performed in clinical suspicion of vasospasm with mFV < 120 cm/s (Kumar et al., 2016).

### Cerebrovascular Reactivity

Cerebrovascular reactivity is attributed to the difference in CBF or CBFV in response to vasoactive stimuli, such as CO_2_, hyper/hypoventilation, acetazolamide (AZ), or breath-holding (Sobczyk et al., 2021). Changes in cerebral perfusion pressure do not affect CBF because cerebral arterioles can control the vascular resistance, which is an important part of autoregulation (Krishnamurthy et al., 2021). CVR impairment is linked to a higher risk of transient ischemic attack and stroke. Furthermore, various clinical disorders affecting the brain microvasculature, such as cerebral autoregulation, vascular dementia, hypertension, and vasospasm have been linked to decreased CVR. CVR examines how CBF responds to exogenous vasoactive stimuli (such as CO_2_ or AZ) and is primarily linked to vasodilation or constriction (Ellis et al., 2016).

There are different methods used for CVR testing, such as CT perfusion, Xenon-enhanced CT, and MR perfusion, however, a common method of CVR testing is the TCD and AZ test (Settakis et al., 2003; Sleight et al., 2021). Hence, TCD velocity is roughly proportional to CBF. Vasodilation reduces the resistance in the main cerebral arteries, which leads to an increase in CBF and CBFV (Piepgras et al., 1990). In reported studies, TCD was performed to evaluate BFV in intracranial arteries using the transtemporal approach by insonating the MCA. According to these studies, the CVR ranges widely among healthy patients tested with TCD and AZ (Naqvi et al., 2013). CVR has been reported to range from 34 to 65%, with velocity increasing from 55–72 cm/s at baseline to 76–97 cm/s post-AZ stimulation. In contrast to the usual reaction of considerably higher velocities after AZ, pathological responses were regarded as reduced CVR (a lesser velocity increase) and exhausted CVR (no velocity increase) (Dahl et al., 1994). In severe clinical conditions, AZ can even produce a “steal phenomenon,” a paradoxical drop in BFV in highly constricted arteries. Arterioles distal to unaffected arteries have normal dilating capability, but arterioles distal to constricted arteries have already reached their maximum dilating capacity and cannot dilate any further. Because blood flow is diverted to (or “taken” by) unaffected arteries, velocities in the constricted artery will be lowered after AZ injection. This type of hemodynamic steal might result in clinical deterioration and increasing cerebral ischemia (Alexandrov et al., 2007). Another study was performed retrospectively on 37 individuals with greater than 50% unilateral carotid stenosis as determined angiographically. TCD was used to calculate the relative change in MFV pre- and post an AZ challenge, and the obtained data was compared to those obtained using single-photon emission computed tomography (SPECT). The CVR of the MCA was dramatically reduced in the carotid stenosis-ipsilateral side. When symptomatic stenosis was present, the CVR impairment was more significant. The degree of carotid stenosis was found to be considerably linked with the CVR. The acquired data indicates that TCD is a valuable technique for assessing CVR since it can provide a variety of clinical information depending on the degree of stenosis (Kim et al., 2005).

### Clinically Diagnostic Limitations of Transcranial Doppler in Cerebrovascular Diseases

Transcranial Doppler has apparent benefits in the treatment of cerebral vascular disorders, but there are also some limitations of this technique. Firstly, the TCD study’s accuracy mostly depends on the TCD operator. In view of this fact, only the best TCD technician or operator should be assigned to the job. Secondly, Anterior TCD has been conducted through the temporal bone for anterior circulation, even though the bone is not always permeable to sound in each patient, particularly in postmenopausal women, where bone *remodeling* produces irregularity of the tabular surface, obviating the ability to return the transmitted signal to the probe (Suri et al., 2011). Furthermore, some individuals, particularly aged individuals, also have not enough bone windows to allow the ultrasound beam to penetrate properly, which could give inaccurate data (Sloan et al., 1989). Age, gender, skull bone thickness, hematocrit value, variation in CO_2_ partial pressure in the blood are the considerable factors that could affect the results (such as velocity) (Tyagi et al., 2000). TCD measures are, however, restricted to the main basal arteries and can only serve as a global rather than local indicator of cerebral BFV (Tyagi et al., 2000).

## Conclusion

In the current study, we explored the role of TCD in cerebrovascular disorders including Cerebral Sinus Venous Thrombosis, acute ischemic stroke, intracranial atherosclerotic stenosis, and Cerebral Vasospasm. TCD ultrasonography provides new insight into the prognosis of cerebrovascular disorders. Furthermore, this technique provides guidance and monitoring for treatment options through rapid evaluation of cerebral hemodynamics. However, TCD imaging is also associated with some limitations, including operator dependency and ultrasound attenuation through the skull and soft tissues. In future, extensive studies are needed to overcome the underlined TCD imaging limitations.

## Author Contributions

YP and YG wrote the manuscript. WW did language editing and helped in the submission process of the manuscript. All authors approved the article for publication.

## Conflict of Interest

The authors declare that the research was conducted in the absence of any commercial or financial relationships that could be construed as a potential conflict of interest.

## Publisher’s Note

All claims expressed in this article are solely those of the authors and do not necessarily represent those of their affiliated organizations, or those of the publisher, the editors and the reviewers. Any product that may be evaluated in this article, or claim that may be made by its manufacturer, is not guaranteed or endorsed by the publisher.
